# Overexpression and Down-Regulation of Barley Lipoxygenase *LOX2.2* Affects Jasmonate-Regulated Genes and Aphid Fecundity

**DOI:** 10.3390/ijms18122765

**Published:** 2017-12-19

**Authors:** Aleksandra Losvik, Lisa Beste, Robert Glinwood, Emelie Ivarson, Jennifer Stephens, Li-Hua Zhu, Lisbeth Jonsson

**Affiliations:** 1 Department of Ecology, Environment and Plant Sciences, Stockholm University, 10691 Stockholm, Sweden; aleksandra.losvik@su.se (A.L.); lisa@sunnerstascience.se (L.B.); 2 Department of Crop Production Ecology, Swedish University of Agricultural Sciences, 75007 Uppsala, Sweden; robert.glinwood@slu.se; 3 Department of Plant Breeding, Swedish University of Agricultural Sciences, 23053 Alnarp, Sweden; emelie.ivarson@slu.se (E.I.); li-hua.zhu@slu.se (L.-H.Z.); 4 Cell and Molecular Science, James Hutton Institute, Invergowrie, Dundee DD2 5DA, UK; jennifer.Stephens@hutton.ac.uk

**Keywords:** aphid resistance, lipoxygenase, *Hordeum vulgare*, *Rhopalosiphum padi*, *Myzus persicae*

## Abstract

Aphids are pests on many crops and depend on plant phloem sap as their food source. In an attempt to find factors improving plant resistance against aphids, we studied the effects of overexpression and down-regulation of the lipoxygenase gene *LOX2.2* in barley (*Hordeum vulgare* L.) on the performance of two aphid species. A specialist, bird cherry-oat aphid (*Rhopalosiphum padi* L.) and a generalist, green peach aphid (*Myzus persicae* Sulzer) were studied. *LOX2.2* overexpressing lines showed up-regulation of some other jasmonic acid (JA)-regulated genes, and antisense lines showed down-regulation of such genes. Overexpression or suppression of *LOX2.2* did not affect aphid settling or the life span on the plants, but in short term fecundity tests, overexpressing plants supported lower aphid numbers and antisense plants higher aphid numbers. The amounts and composition of released volatile organic compounds did not differ between control and *LOX2.2* overexpressing lines. Up-regulation of genes was similar for both aphid species. The results suggest that *LOX2.2* plays a role in the activation of JA-mediated responses and indicates the involvement of *LOX2.2* in basic defense responses.

## 1. Introduction

Many crop species suffer considerable damage due to infestation by aphids. Although they cause little wounding injury to the plant tissue, they can induce stunting, chlorosis, necrosis, or plant deformation [1,2]. Nutrient withdrawal at high population densities, together with the transmission of plant viruses, contributes to losses in yield. Bird cherry-oat aphid (BCA; *Rhopalosiphum padi* L.) is a common pest on cereals, including barley and wheat. Although it does not cause visible injury, it is a vector for barley yellow dwarf virus, which causes symptoms such as leaf discoloration and dwarfing [3,4,5]. Attempts to breed for aphid resistance have not produced any commercial barley cultivar resistant to BCA; however, they have resulted in a number of doubled haploid breeding lines with moderate resistance to this aphid species [6,7]. The source of resistance in these lines is an accession of wild barley *Hordeum vulgare* L. ssp. *spontaneum* (Hsp5), and the resistance is manifested as decreased (up to 50%) nymphal growth [6]. Hsp5, together with a doubled haploid line DH28:4 (full number 5172-28:4), and two unrelated susceptible cultivars were used in a comparative transcript profiling study, which resulted in the identification of four genes induced by BCA only in the resistant genotypes [8]. Based on this study, we currently test the hypothesis that the increased resistance might be attributed to the up-regulation of those specific genes.

One of the contigs that showed upregulation on aphid infestation in the two moderately resistant barley genotypes, but not in the two susceptible cultivars, was contig2305_at. This contig corresponds to a gene with the GenBank accession no AJ507212, with the annotation *Lox2:Hv:2* (or *LOX2.2*). The gene belongs to a group of three barley lipoxygenase genes (*Lox2:Hv:1*; *Lox2:Hv:2;* and *Lox2:Hv:3*) which were characterized as chloroplastic 13-lipoxygenase (13-LOX) genes [9,10]. Plant lipoxygenases (LOXs) catalyze the oxidation of polyunsaturated fatty acids, thereby generating hydroperoxy fatty acids. The two major substrates in plant membranes are linoleic acid, containing 18 carbons and two double bonds (C18:2), and linolenic acid (C18:3), with three double bonds. In leaf tissue, they may account for 60 to 70% of the fatty acids in membranes [11,12]. They are particularly abundant in the chloroplast membranes, where they are esterified to the chloroplast lipids monogalactosyl diacylglycerol and digalactosyl diacylglycerol, but they are also constituents of other cell membranes, esterified to phospholipids such as phosphatidylcholine and phosphatidylethanolamine [11].

LOXs have been divided into two types; type 1-LOXs without a transit peptide and a high (>75%) sequence similarity, and type 2-LOXs with a chloroplast transit peptide and a moderate sequence homology to other LOXs (>35%) [13,14,15]. Another basis for classification is the position of the oxidation of the fatty acid substrate, linoleic acid. The oxidation may be at either position 9 or 13, resulting in 9-LOXs or 13-LOXs. Generally, type 2-LOXs have been shown to exhibit 13-LOX activity and type 1-LOXs to exhibit 9-LOX activity, but there are examples of LOX enzymes without this pattern of specificity or with dual position specificity [14,16]. The barley *LOX2.2* gene was cloned and sequenced and found to contain a putative chloroplast target peptide and, based on this, was classified as a type 2-LOX [10]. Assays of the recombinant enzyme with linoleic acid showed that the products were 13(S)-hydroperoxy fatty acids [10], and LOX2.2 has since been characterized as a chloroplastic 13-LOX.

Downstream of plant LOXs acting on linoleic and linolenic acid, there are two biosynthetic pathways with end products of significance for plant defense against insects. In one branch of this pathway, allene oxide synthase (AOS) acts on the hydroperoxy fatty acid(s) and subsequent steps lead to the synthesis of jasmonic acid (JA) [17,18,19,20]. In barley, three AOS sequences have been identified [21,22]. Two of them, AOS1 and AOS2, can use both 9- and 13-hydroperoxides as substrates and were found localized in chloroplasts [21], and the third, AOS3, could use only 13-hydroperoxides [22]. In the other branch, hydroperoxide lyase (HPL) catalyzes steps leading to green leaf volatiles (GLVs), C6-aldehydes, alcohols, and their esters [23,24]. HPL is localized in the chloroplasts in a tomato [25] and the analysis of an HPL sequence from barley predicted that it might contain a transit peptide directing it to plastids [26]. GLVs have been shown to induce defense responses and are involved in indirect defense in plant-insect interactions [20,23,27,28].

Functional genomics has been applied to the study of 9- or 13-LOXs in several plant species. This has provided information on their effects on pathogens and insects. With reference to the type of interaction and plant tissue investigated in the current study, insects on monocot leaves, several reported findings are relevant. A rice LOX, encoded by *OsLOX1*, was localized to chloroplasts and had dual specificity with both C-9 and C-13 oxidized products [16]. Two lines overexpressing *OsLOX1* showed reduced plant mortality when infested with the phloem-feeding brown plant hopper (*Nilaparvata lugens* Stål). These lines had higher endogenous levels of JA and (Z)-3-hexenal in response to an attack by the brown plant hopper [16]. Another LOX in rice, with putative 13-LOX activity, *OsHI-LOX*, was cloned and found to be localized to chloroplasts [29]. Silencing of this gene reduced the levels of insect-induced JA, but there were no effects on wound-induced (Z)-3-hexenal or (Z)-3-hexenol production. The antisense plants were more susceptible to chewing larva from the striped stem borer (*Chilo suppressalis* Walker) and the rice leaf folder (*Cnaphalocrocis medinalis* Guenée), but more resistant to the brown plant hopper [29]. The same research group studied a chloroplast-localized 9-LOX in rice, *Osr9-LOX1* [30]. Antisense expression of the sequence increased the amounts of insect-induced JA and reduced levels of wound-induced (Z)-3-hexenal. The antisense plants showed an increased resistance to striped stem borer larvae, but the survival rate of nymphs of the brown plant hopper was higher on these plants [30]. Thus, in rice, the activity of LOX’s with 9- or double 9,13-specificity and channeling substrates into GLV production (*Osr9-LOX1; OsLOX1*) were detrimental for the phloem-feeding brown plant hopper, whereas a 13-LOX involved in JA biosynthesis (*OsHI-LOX*) appeared to benefit this insect. The negative effect of GLVs on the brown plant hopper was supported by studies of a rice hydroperoxide lyase gene *OsHPL3* [31]. OsHPL3 was shown to be involved in the production of GLVs and brown plant hopper performed better on *hpl3-1* mutants and less well on overexpressing transformants [31]. In maize, one 13-LOX involved in resistance against chewing *Spodoptera exigua* Hübner larvae, *ZmLOX10*, has been reported [32]. ZmLOX10 was found to be localized outside of chloroplasts and *lox10* mutants showed more damage by *S. exigua* larvae than wild type plants [32].

The hypothesis tested in the current study is that the upregulation of *LOX2.2* by BCA in moderately resistant barley genotypes indicates that the gene mediates efficient defense against this aphid. Based on the characterization of barley *LOX2.2* as a 13-LOX with a putative plastid target sequence, the gene might be involved in the production of either JA or GLVs. In either case, we expected that transformed plants overexpressing *LOX2.2* would be more resistant to aphids and antisense plants more susceptible. However, since it is not known whether other signal pathways may be affected, this outcome might be compromised, as shown in the rice *OsHI-LOX* studies [29]. In order to detect possible differences in effects towards generalist and specialist aphids, the aphid tests were carried out with both BCA and the generalist green peach aphid (*Myzus persicae* Sulzer) (GPA).

## 2. Results

### 2.1. Phenotypes and Transcript Abundance of Jasmonic Acid (JA)-Regulated Genes

**Figure 1:**
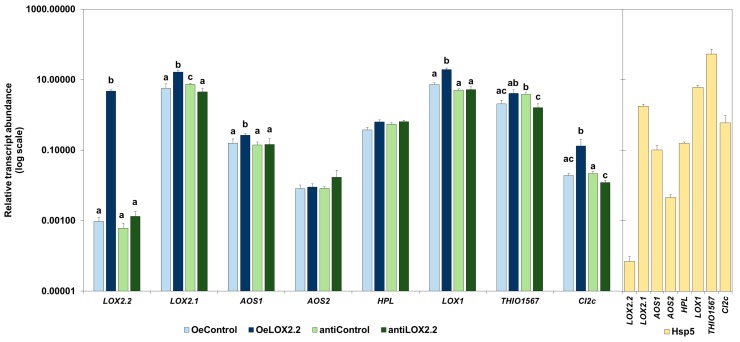
Relative transcript abundance of *LOX2.2* and a selection of other jasmonic acid (JA)-regulated genes in OeLOX2.2 and antiLOX2.2 lines, their respective controls, and Hsp5 plants. Error bars indicate SE. Letters indicate significant difference between lines for a particular gene (Kruskal-Wallis test at *p* < 0.05). Samples were from leaves of nine-day old plants (Hsp5, primary leaf) or 14-day old plants (all other genotypes, second leaf). Reference genes: *Hsp70* and *Tubulin*.

For experiments with aphids, we selected one line with constitutive transgene overexpression (OeLOX2.2) and one line transformed with an antisense construct (antiLOX2.2) (Figure S1). There were no differences in shoot fresh weight or length, or any other obvious phenotypic differences between the transgenic lines and the control plants (Figure S2). We first studied the constitutive transcript abundance of *LOX2.2* and a selection of other JA-regulated genes in uninfested plants. The OeLOX2.2 line had a clearly higher transcript abundance of *LOX2.2* (*p* < 0.0001) compared to its control (Figure 1). In addition, four other genes known to be regulated by JA showed a significantly higher transcript abundance in the overexpressing plants compared to the control plants: *LOX2.1* (*p* < 0.0001), *AOS1* (*p* < 0.05), *LOX1* (*p* < 0.0001), and *CI2c* (*p* < 0.01) (Figure 1). We found no significant difference between the antiLOX2.2 line and its control with regard to the transcript abundance of *LOX2.2*. Three of the genes regulated by JA: *LOX2.1* (*p* < 0.05), *THIO1567* (*p* < 0.05), and *CI2c* (*p* < 0.05) exhibited a significantly lower transcript abundance in the antiLOX2.2 plants than in the controls (Figure 1).

We compared the transcript abundance of JA-regulated genes in the moderately resistant wild barley Hsp5 from the background study with the currently studied azygous controls and transgenic plants. The data were not included in the statistical analysis of significant differences, because the plant tissue and age were not identical. They showed a higher transcript abundance of *THIO1567* and *CI2c* in Hsp5 compared to in the other genotypes, and a lower abundance of *LOX2.2* (Figure 1). A general observation was that the transcript abundance of *LOX2.2* was much lower than that of *LOX2.1* in all genotypes (except in the OeLOX2.2 line) (Figure 1).

### 2.2. Volatile Composition with and without Aphids in LOX2.2 Overexpressing Plants

**Figure 2:**
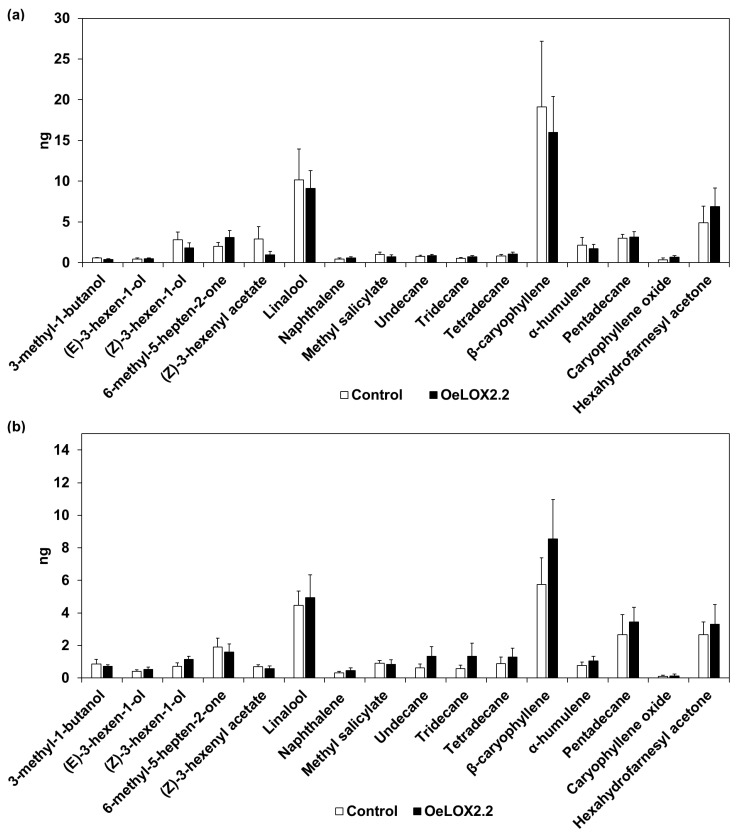
Volatile profiles of control and OeLOX2.2 lines. (**a**) Uninfested plants, (**b**) bird cherry-oat aphid (BCA)-infested plants. White bars represent the control plants and black bars the overexpressing line OeLOX2.2. Average amounts in ng (±SE) in 48 h volatile collection from 15 plants (*n* = 6).

The OeLOX2.2 line and its control line were infested by BCA for five days and volatile profiles of infested and uninfested plants were analyzed (Figure 2). The major volatiles were β-caryophyllene and linalool, which are both synthesized via the isoprenoid pathway. Volatiles derived from fatty acids via the lipoxygenase pathway were (E)-3-hexen-1-ol, (Z)-3-hexen-1-ol, and (Z)-3-hexenyl acetate. The signal compound methyl salicylate was detected in the volatile blend at low amounts. Differences in the amounts of the above compounds were analyzed using two-way ANOVA with “line” and “aphids” as fixed factors. There were no differences between the lines (Table 1). The amounts of β-caryophyllene and linalool were significantly lower in aphid-infested plants compared to uninfested plants (*p* < 0.05, but with no significant difference in the post hoc analyses) (Table 1). The total amounts of LOX-derived volatiles were also lower from aphid-infested plants (*p* < 0.05), and the post-hoc analysis showed a significant difference (*p* < 0.05), specifically for the control line (Table 1). The main contribution to this difference was (Z)-3-hexen-1-ol, whereas the amounts of (E)-3-hexen-1-ol and (Z)-3-hexenyl acetate did not differ between aphid-infested and uninfested plants (Table 1). There were no significant differences in the amounts of methyl salicylate related to either aphid infestation or to line.

### 2.3. Bird Cherry-Oat Aphid (BCA) and Green Peach Aphid (GPA) Settling, Fecundity, and Lifespan on LOX2.2 Transformed Plants

**Figure 3:**
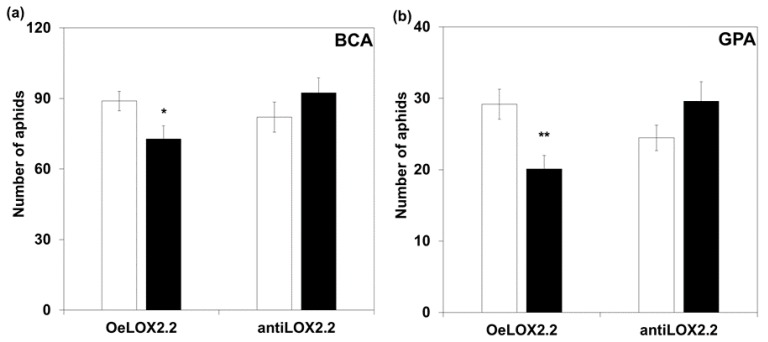
Aphid numbers on control and transgenic lines, five days after infestation with twenty adult apterous aphids. (**a**) Numbers of bird cherry-oat aphid (BCA), (**b**) numbers of green peach aphid (GPA). White and black bars indicate, respectively, control and transgenic lines. Bars indicate the average (±SE). *n* = 12 for BCA and *n* = 22 for GPA. Asterisks indicate significant differences after aphid infestation between control and transgenic plants (*t*-test, * *p* ≤ 0.05; ** *p* ≤ 0.01).

Results from aphid settling in a no-choice test showed no significant differences between control and transgenic plants in the number of aphids settled at any of the time points 2, 4, or 6 h (Figure S3). Aphid fecundity was significantly lower on OeLOX2.2 compared to its control plants (for BCA *p* < 0.05, and for GPA *p* < 0.01, *t*-test) and there was a tendency for higher aphid numbers on the antiLOX2.2 plants compared to the control (Figure 3). The life span tests did not reveal any significant differences between the transformed plants and their controls for any of the parameters of life span and fecundity, neither for BCA nor for GPA (Table 2).

### 2.4. Transcript Abundance of Genes Regulated by JA in Aphid Infested Tissue

**Figure 4:**
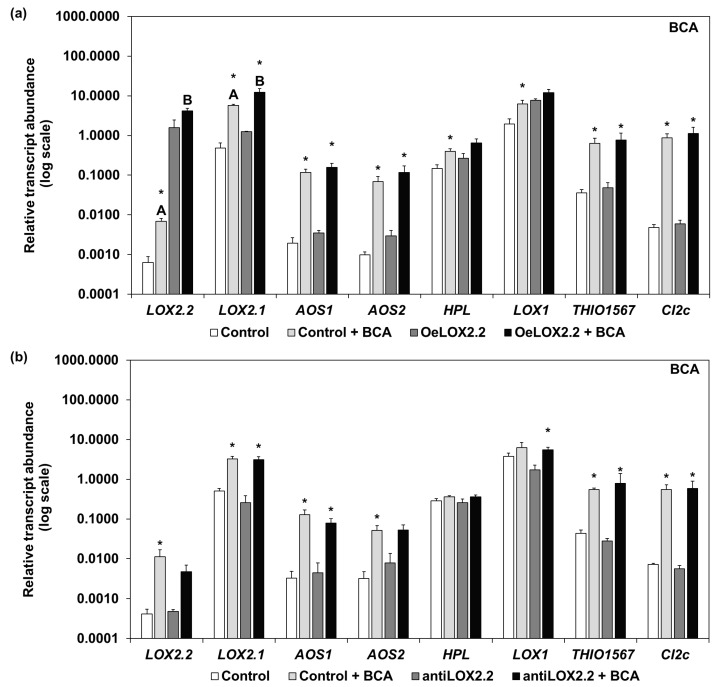
Relative transcript abundance in control and transgenic lines without aphids and infested with BCA for five days. (**a**) Control and OeLOX2.2 plants, (**b**) control and antiLOX2.2 plants. White and light grey bars represent control plants, with and without aphids; dark grey and black bars represent transgenic lines with and without aphids. The transcript abundance is relative to reference genes *Hsp70* and *Tubulin* (±SE). Different letters indicate significant differences between the infested lines, asterisks indicate significant differences for the same line with and without BCA (* *p* < 0.05 Mann-Whitney test; *n* = 3 for plants without aphids, *n* = 4 for infested plants; three technical replicates).

**Figure 5:**
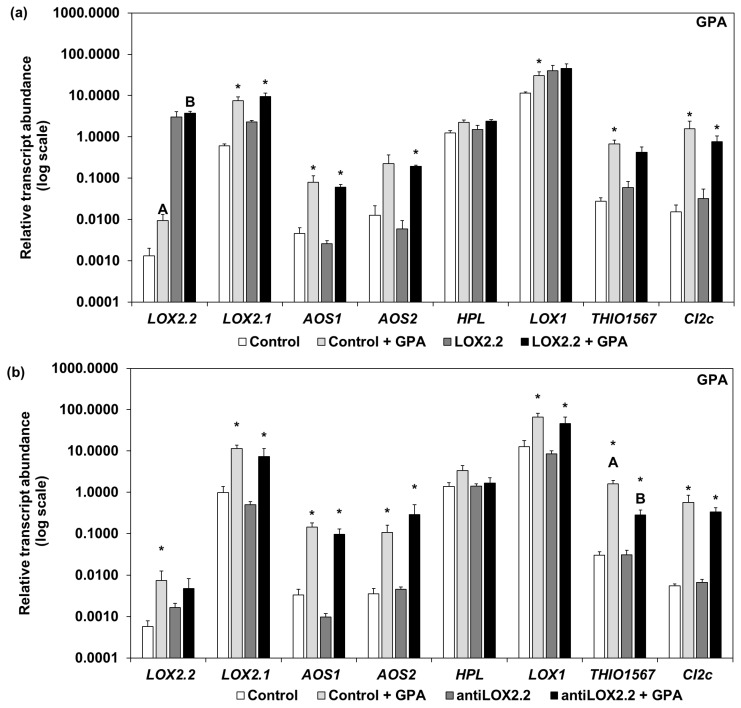
Relative transcript abundance in control and transgenic lines without aphids and infested with GPA for five days. (**a**) Control and OeLOX2.2 plants, (**b**) control and antiLOX2.2 plants. White and light grey bars represent control plants, with and without aphids; dark grey and black bars represent transgenic lines with and without aphids. The transcript abundance is relative to reference genes *Hsp70* and *Tubulin* (±SE). Different letters indicate significant differences between the infested lines, asterisks indicate significant differences for the same line with and without GPA (* *p* < 0.05 Mann-Whitney test; *n* = 3 for plants without aphids, *n* = 4 for infested plants; three technical replicates).

The relative transcript abundance of selected genes was studied in plants infested with BCA (Figure 4) or GPA (Figure 5) for five days. Comparing gene expression between uninfested and BCA-infested OeLOX2.2 and control plants, most genes showed a higher transcript abundance with BCA than without, the only exceptions being *LOX2.2*, *HPL*, and *LOX1* on OeLOX2.2 (Figure 4a). With regard to control and antiLOX2.2 plants, most genes had a higher transcript abundance in BCA-infested plants, but with *HPL* and *LOX1* in the control plants and *LOX2.2*, *AOS2*, and *HPL* in the antisense plants as exceptions (Figure 4b). Comparing gene expression in the BCA-infested plants between control and transgenic lines, both *LOX2.2* and *LOX.2.1* showed a higher transcript abundance in OeLOX2.2 than its control (Figure 4a), despite lower numbers of BCA on OeLOX2.2 (an average ±SD of 63 ± 18) than on the control (76 ± 5) on the four plants examined in this study. There were no significant differences in transcript abundance between the control and antisense line in aphid-infested plants (Figure 4b). The mean number of BCA on the four studied antisense plants was 72 ± 12 (±SD) compared to 62 ± 8 on the control plants.

Infestations with GPA gave similar results as BCA for the levels of gene expression and the pattern of up-regulation (Figure 5). Considering the overexpressing line and its control, all genes were found at a higher expression with GPA than without, except *LOX2.2* and *HPL* (neither in OeLOX2.2 nor the control), and *THIO1567* in OeLOX2.2 (Figure 5a). The experiment with the antisense line and its control showed all genes except *LOX2.2* in the antisense line and *HPL* in both lines to be at higher expression levels with GPA than without (Figure 5b). Comparing gene expression between the lines in the GPA-infested tissue, *LOX2.2* was at higher expression levels in OeLox2.2 (Figure 5a) and *THIO1567* at lower expression levels in the antisense line (Figure 5b), compared to their respective aphid-infested controls. The mean number of aphids ±SD on the plants studied was 29 ± 9 on the control compared to 21 ± 6 on OeLOX2.2, and 20 ± 6 on the control compared to 35 ± 4 on the antiLOX2.2 plants.

The effects of BCA infestation on the expression of the same gene sequences were also examined in the two moderately aphid-resistant barley genotypes used in the background study, Hsp5 and DH28:4 (Figure S4). The conditions were the same as in the original study. The data confirm and extend the earlier results [8]. All studied gene sequences were significantly up-regulated in both genotypes after BCA infestation, except *THIO1567* in Hsp5 (Figure S4).

## 3. Discussion

Previous studies on LOXs in barley leaves have shown that LOX2.1 is the major LOX [10,33]. In comparison, *LOX2.2* is present both at much lower levels at the transcript level [10] and as a protein [10,33]. This relationship was confirmed in the current study, and could be quantified at the transcript level. We found that modifying the expression of *LOX2.2* influenced the relative transcript abundance of other genes known to be regulated by JA [34,35], such that they were higher in the overexpressing plants and lower in the antisense plants.

The overexpression of *LOX2.2* in the OeLOX2.2 line was clearly confirmed in our plants as a significantly higher expression of *LOX.2.2* and higher expression of a number of other JA-regulated genes (Figure 1, Figure 4 and Figure 5). This indicates that the level of expression of *LOX2.2* influences other genes. *LOX2.2* had a low expression both in the control and antisense lines; however, we were able to indirectly confirm the antisense status by showing a lower expression of three other JA-regulated genes (Figure 1), and no significant upregulation of *LOX2.2* by aphids (Figure 4 and Figure 5) in this line.

The effects of modifying *LOX2.2* via overexpression or silencing had low or moderate effects on the performance of both BCA and GPA. There were no indications that aphid host acceptance was affected on plants with modified *LOX2.2* expression compared to their controls (Figure S3). Further tests did not reveal any difference in the parameters of life span and fecundity when individual nymphs were observed throughout their life span. This lack of long-term effect on aphids that have established phloem feeding suggests that any differences in phloem composition in the transgenic plants do not affect aphid biology.

To explain the effects on fecundity in five-day tests (Figure 3), we suggest that proteins or protein inhibitors with an inhibitory effect on aphid metabolism and/or reproductive functions are present at higher or lower levels, respectively, in the over-expressing and antisense *LOX2.2* transformants. Two types of such proteins were previously proposed to have defense functions against aphids in barley; thionins and proteinase inhibitors [7,8]. The present study adds suggestive evidence to these ideas. Constitutive expression of the thionin gene *THIO1567* was lower in the antisense line and that of the proteinase inhibitor *CI2c* was higher in OeLOX2.2 and lower in antiLOX2.2 compared to their respective controls (Figure 1). In addition, *THIO1567* was at a lower expression in the antisense line compared to the control line upon GPA infestation (Figure 5), and both *THIO1567* and *CI2c* were found at a higher transcript abundance in the resistant wild barley Hsp5 than in the other genotypes (Figure 1). The proteinase inhibitor, *CI2c*, was previously shown to inhibit GPA reproduction when expressed in Arabidopsis [36]. Thionins are sulfur-rich small proteins with in vitro toxicity against plant fungal pathogens and bacteria [37,38]. Barley contains leaf thionins, found both intracellularly and in the cell wall [39], and it is likely that aphids encounter these small proteins when penetrating the tissue intercellularly and when probing intracellularly.

*LOX2.1* constitutive transcript abundance was affected in transgenic plants. It was higher in the OeLOX2.2 line and lower in the antisense line (Figure 1). This suggests that products arising from the biosynthetic step where the LOX2.2 protein is involved regulate the expression of *LOX2.1*. A role for *LOX2.2* in the regulation of other genes is in accordance with previous findings from the treatment of floating barley leaves with JA- or JA-isoleucine conjugate [10]. The treatments resulted in higher levels of *LOX2.2* mRNA at 1 h after treatment, the earliest time point measured, whereas increased mRNA of *LOX2.1* and *LOX2.3* was clearly exhibited later, the first shown at 2 h [10]. Thus, it is possible that up-regulation of *LOX2.2* is a pre-requisite for the up-regulation of the other *LOX* genes.

The modified expression of other genes known to be regulated by JA in barley suggests that the product downstream of LOX2.2 involved in regulation is JA. However, it has been shown that C6 volatiles derived from the HPL pathway may induce largely the same genes as methyl jasmonate. This was shown in Arabidopsis for (E)-2-hexenal [40], (Z)-3-hexenal, and (Z)-3-hexenol [41,42]. In maize, it was shown for (Z)-3-hexenol [43,44], (E)-2-hexenal, and (E)-3-hexenal [44]. Our analysis of volatiles did not reveal any difference between the overexpressing line and its control line in the amounts of (E)-3-hexen-1-ol, (Z)-3-hexen-1-ol, or (Z)-3-hexenyl acetate collected during 48 h. This does not support a role for *LOX2.2* in increased GLV production. It should be noted, however, that LOX derived volatiles, especially (Z)-3-hexen-1-ol, were reduced after aphid infestation in the control line but not in the overexpressing line (Table 1). Further, it is possible that the amounts needed to affect gene regulation are very low and were not detected in our analyses. It was earlier reported that an endogenous increase in JA caused by sorbitol osmotic stress does not induce *LOX2.1* [9], which supports the idea of GLVs as inducing factors. The suggested function of *LOX2.2* in barley would in this case be analogous to that of *LOX2* in Arabidopsis, which is required for wound-induced JA accumulation [45]. Recently, Arabidopsis *LOX2* was shown to be necessary for the synthesis of GLVs after wounding [46], suggesting that GLVs may act as signal molecules in promoting JA biosynthesis. A similar system is found in maize, where it has been reported that *ZmLOX10* is involved in the production of GLVs and is needed for the wound-induced production of JA mediated by *ZmLOX8* [32]. A difference compared to barley and Arabidopsis is that *ZmLOX10* is localized outside of chloroplasts [32]. Our data do not provide conclusive evidence for whether GLVs are involved in signaling and this aspect needs further investigation.

There is evidence that JA-regulated genes are involved in efficient defense against phloem feeding insects ([47] and references therein). *LOX*s are reported to be upregulated by aphids in a number of plant-aphid interactions (reviewed in [48]), but there are few studies with a functional genomics approach to investigate the role of these enzymes in plant resistance towards aphids. An interesting exception is the study of *LOX5* in Arabidopsis. This gene was found to be upregulated in roots by GPA in shoots and its expression resulted in the production of oxylipins that were transferred to the shoot where they promoted GPA performance [49]. GPA was also studied in Arabidopsis, with genotypes modified in biosynthetic steps downstream of LOX, and as a consequence, lacking JA or aldehydes [50]. GPA preferred the *aos-hpl* genotype in choice tests and had a clearly higher fecundity on this genotype compared to the trichomeless background (*gl-1*, accession Col-0). The interpretation is however not clear-cut with regard to the role of different signal compounds, since *aos-hpl* lacked both C6 aldehydes, JA and camalexin [50], and camalexin has been shown to play a role in defense against GPA in Arabidopsis [51].

Our study gives little support to the idea of JA-mediated defense being efficient towards aphids in barley. We found several JA regulated genes expressed at higher transcript levels in the overexpressing *LOX2.2* line and three such genes were lower in the antiLOX2.2 line, but the effect on aphid performance was low to moderate. It should be noted that the fecundity of GPA on barley is much lower than that of BCA. Nevertheless, the effect of modifying *LOX2.2* was similar towards the specialist BCA and the generalist GPA. This does not support a critical role for the studied genes in the non-host character of barley towards GPA. Instead, it seems that the genes studied are involved in basic defense responses, which hinder both specialist and generalist aphids, but do not prevent their successful proliferation.

## 4. Materials and Methods

### 4.1. Aphid Rearing

Individuals of BCA, *R. padi* L. and GPA, *M. persicae* Sulzer were collected in the field near Uppsala, Sweden. Aphid populations of parthenogenetically reproducing females were reared for several years on oat (*Avena sativa* L., cv. Kerstin) (BCA) and kohlrabi (*Brassica oleracea* L., cv. Delikatess weisser) (GPA). They were reared in a growth chamber at 22 °C, 50% humidity, and 150 μmol photons m^−2^·s^−1^, with a photoperiod of 16 h light/8 h darkness.

### 4.2. Plant Cultivation

Seeds of barley (*H. vulgare* L.) cv. Golden Promise were sown in pots (7 cm × 7 cm) filled with planting soil (Plugg-och Såjord, Weibulls, Sweden) and transferred to a growth chamber with conditions as described above for aphid rearing. For experiments with aphids, plants were 14 days old, unless mentioned otherwise.

### 4.3. Plasmid Constructs, Plant Transformation and Selection

For *LOX2.2* overexpression and antisense transformation, RNA was extracted, respectively, from the barley doubled haploid breeding line 5172-28:4 (DH28:4) and barley cv. Golden Promise, using the NucleoSpin^®^ RNAII kit (Macherey-Nagel, Düren, Germany) following the manufacturer’s instructions. Three micrograms of RNA were used for the synthesis of first strand cDNA using the Transcriptor High Fidelity cDNA Synthesis Kit (Roche) according to the manufacturer’s instructions. The ORF of a barley *LOX2.2* encoding gene used in the overexpressing transformants was amplified from the cDNA using the primers 5′-CACCATGCAGACGGCAACCAAGCCT-3′ and 5′-CCGAACAGCATTTCCATTTAATCAGAATG-3′. For barley antisense transformation, an *LOX2.2* fragment (cloned from Golden Promise) was expressed in the antisense direction (Figure S1). For transformation, Platinum Taq High Fidelity DNA polymerase (Invitrogen) was used for the PCR reaction at 94 °C for 30 s, 30 cycles at 94 °C for 15 s, 55 °C for 15 s, and 68 °C for 1 min, followed by 68 °C for 7 min. The PCR product was cloned into the Gateway^®^ pCR8/GW/TOPO cloning vector (Invitrogen) according to the manufacturer’s instructions and introduced by *att* site recombination into the destination vector pBract 214 (provided by Dr Mark Smedley, John Innes Centre, http://www.bract.org). The binary plasmid was co-transformed into *Agrobacterium tumefaciens* strain AGL1, together with helper plasmid pSoup [52], and used to transform barley immature embryos as described in [53]. Barley transformants were selected on medium containing hygromycin (50 μg·mL^−1^, Sigma-Aldrich, St. Louis, MO, USA), and analyses were performed on T2 or T3 lines homozygous for a single-gene insertion. Controls were azygous lines selected at T2.

### 4.4. RNA Extraction and RT-qPCR

For RT-qPCR studies, plant material was frozen in liquid nitrogen and stored at −80 °C until being used for RNA extraction. For the data presented in Figure 1, the mid-section of the second leaf was used. For data comparing plants with and without aphids, part of the leaf within the aphid cage was used from four aphid-infested and four control plants at day five of the fecundity test (see below). RNA isolation, RT-qPCR conditions, and calculations of relative transcript accumulation were carried out as described in [7]. *Hsp70*, *Tubulin*, and *SF427* were used as reference genes. The primer sequences are shown in Table S1.

### 4.5. Volatile Collection and Analysis

Charcoal filters and glass tubes containing molecular absorbent (Porapak Q, 50 mg) were baked overnight under nitrogen flow at 180 °C and 140 °C, respectively. PET (polyethylene terephthalate) bags (35 × 43 cm; Melitta Scandinavia, Helsingborg, Sweden) and foil were baked for 2 h at 140 °C. Entire pots containing 15 plants were placed inside the PET bags and then open end sealed. Each bag contained a single pot. Soil in plant pots was covered with aluminum foil and two glass rods were placed in the soil to prevent the collection bags touching the leaves. Charcoal-filtered air was pushed in from the bottom of the bag at 600 mL per min and pulled out through a glass tube containing Porapak at 400 mL per min, positioned at the top.

Volatiles from BCA-infested control and OeLOX2.2 plants were collected by air-entrainment after five days of infestation. Each pot containing 15 plants was infested with 150 aphids at day 1, resulting in an average of 10 aphids/plant. The collection was for 48 h since it has been previously shown that this period was optimal for collecting sufficient amounts of barley volatiles for analysis [54]. After 48 h, Porapak tubes were eluted with 750 μL redistilled dichloromethane (Sigma-Aldrich, St. Louis, MO, USA) and the eluted solvent was concentrated to 50 μL under a gentle nitrogen flow. An internal standard (1-nonene) was added to the eluted sample before concentration to achieve a concentration of 1.8 ng·μL^−1^ in the final sample. Six replicates (pots with 15 plants each) were used for each experimental treatment.

Plant volatiles were analyzed using coupled gas chromatography (GC)/mass spectrometry. A 3 μL aliquot of the entrained sample was injected into an Agilent 7890A GC (Agilent Technologies, Santa Clara, CA, USA) equipped with a cold-on-column injector and fitted with an HP-1 column (30 m, 0.25 mm i.d., and 0.25 μm film thickness; J & W Scientific, Folsom, CA, USA) coupled to an Agilent 5975C mass selective detector (electron impact 70 eV, 230 °C). The GC program was set to start at 30 °C for 4 min, and set to rise 5 °C min^−1^ to 150 °C, then at 10 °C min^−1^ to 250 °C. The carrier gas was helium with a flow rate of 1 mL per min. Volatile compounds were identified by comparing the mass spectra and retention indices against a commercial library (NIST 08; http://www.nist.gov/srd/) and commercial authentic standards. Quantifications were made using quantification curves constructed with authentic standards.

### 4.6. Biological Tests with Aphids

Aphid settling was assessed in a no-choice test. A small cage (5 cm long, 2.5 cm diameter) was mounted on the second leaf of each control and transgenic plant growing in separate pots. Ten adult apterous aphids were transferred to each cage which was sealed with a sponge for the duration of the experiment. The plant pots were placed in a transparent 4 L cage (10 cm × 10 cm × 40 cm) and kept under growth conditions as described for aphid rearing. The number of settled aphids was counted after 2, 4, and 6 h. Eight replicates were performed.

In five-day fecundity experiments, 20 adult apterous aphids were carefully placed within a small plastic cage (see aphid settling) mounted on the mid-section of the second leaf of a 14-day old plant. The cage was sealed with a sponge. After 3 h, the cage was opened to allow for air circulation and prevent the build-up of ethylene. Each plant was placed in a 4 L polycarbonate cage and kept at growth conditions as described under aphid rearing. The total number of aphids was counted after five days. For BCA, two repetitions, each with six plants, were carried out. GPA shows higher variability and three repetitions were carried out, with six, six, and 10 plants. Experiments with BCA-infestation on Hsp5 and DH28:4 were carried out the same way, except that the plants were seven days old at the start, the small cage was mounted on the first leaf, the infestation was for 48 h, the small cage stayed closed, and *n* = 6. Control plants had empty cages mounted on the corresponding leaf. 

In life-span experiments, one apterous adult BCA or GPA was placed within the small cage, as described above. When an adult produced its first offspring, the adult and all but one nymph were removed. The reproduction of this nymph was monitored during its life span, by daily counting and the removing of newborn nymphs. The intrinsic rate of population increase (*r_m_*) was calculated using a formula by Wyatt and White (1977) as 0.738 (ln Nd)/d, where Nd is the number of progeny produced by an aphid in a period equal to the pre-reproductive time and d is the pre-reproductive time in days [55].

### 4.7. Statistical Analysis

Normal distribution of data was analyzed using the Kolmogorov-Smirnov and Shapiro-Wilk tests and was confirmed for fecundity data during five-day tests, as well as volatile data, but not for aphid settling, life span, or relative transcript abundances. Analysis of differences in fecundity was performed using a *t*-test at *p* < 0.05. Differences in volatile amounts were analyzed using two-way ANOVA (at *p* < 0.05) followed by Tukey B as a post-hoc test. Differences in settling or transcript abundance were analyzed using a Mann-Whitney test at *p* < 0.05 or a Kruskal-Wallis test followed by a Conover test as post hoc analysis at *p* < 0.05. Results from the life span experiment were analyzed using a Kruskal-Wallis test at *p* ≤ 0.05. All statistical analyses were performed with StatPlus Pro v5 or v6 for Windows from AnalystSoft Inc. or at astatsa.com [56].

## 5. Conclusions

Modifying the expression of *LOX2.2* affects the expression of other JA-regulated genes, notably *LOX2.1*, indicating that products arising from LOX2.2 activity are involved in their regulation. Overexpression or antisense suppression of *LOX2.2* has no effect on settling or the life span of the specialist aphid BCA or the generalist aphid GPA, but the short-term fecundity of both aphids is lower on *LOX2.2* over-expressing plants and higher on antisenseLOX2.2 plants. In view of the moderate effects on aphids of modifying the expression of a number of JA-regulated genes, this study gives little support to the idea that JA-regulated genes are involved in efficient defense against aphids in barley.

## Figures and Tables

**Table 1:** Significant differences in volatile amounts from the barley OeLOX2.2 line and its control line with and without BCA. Volatiles were collected during 48 h. Analysis by two-way ANOVA with “line” and “aphids” as fixed factors, followed by Tukey B as a post-hoc test (*n* = 6).

**Comparisons between Plants with and without BCA**	**Difference**	***p*-Value**
Sum of LOX derived volatiles: (E)-3-hexen-1-ol, (Z)-3-hexen-1-ol and (Z)-3-hexenyl acetate		0.049
(Z)-3-hexen-1-ol		0.029
β-Caryophyllene		0.044
Linalool		0.046
**Comparisons within control line with and without BCA**		
Sum of LOX derived volatiles: (E)-3-hexen-1-ol, (Z)-3-hexen-1-ol and (Z)-3-hexenyl acetate	−4.34	0.028
(Z)-3-hexen-1-ol	−2.07	0.022

**Table 2:** Lifespan and reproduction of BCA and GPA on OeLOX2.2, antiLOX2.2, and their respective controls. The results represent mean values (±SE). *r*_m_ = intrinsic rate of population increase. There were no significant differences between control and transgenic lines for any of the parameters (*p* > 0.05, Kruskal-Wallis test).

**BCA**	**Control (*n* = 6)**	**OeLOX2.2 (*n* = 6)**	**Control (*n* = 6)**	**antiLOX2.2 (*n* = 6)**
Pre-reproductive days	6.0 ± 0.0	6.2 ± 0.2	6.2 ± 0.2	6.2 ± 0.2
Number of nymphs/aphid	58.7 ± 2.3	60.0 ± 5.1	52.2 ± 6.1	57.8 ± 3.4
Nymphs/reproductive day	4.95 ± 0.29	4.56 ± 0.29	4.55 ± 0.57	5.07 ± 0.34
Reproductive life (days)	12.0 ± 0.6	13.2 ± 0.9	11.5 ± 0.2	11.5 ± 0.6
Life span (days)	26.8 ± 1.8	29.2 ± 2.3	26.8 ± 1.7	30.3 ± 1.7
*r*_m_	0.43 ± 0.01	0.41 ± 0.012	0.41 ± 0.022	0.42 ± 0.008
**GPA**	**Control (*n* = 8)**	**OeLOX2.2 (*n* = 5)**	**Control (*n* = 6)**	**antiLOX2.2 (*n* = 8)**
Pre-reproductive days	10.3 ± 0.6	10.8 ± 0.7	12.0 ± 0.6	11.6 ± 0.6
Number of nymphs/aphid	17.7 ± 3.0	18.2 ± 2.4	17.8 ± 1.2	18.3 ± 3.2
Nymphs/reproductive day	1.03 ± 0.11	1.06 ± 0.11	1.0 ± 0.1	0.97 ± 0.13
Reproductive life (days)	16.9 ± 1.5	17.0 ± 0.9	18.2 ± 1.1	18.0 ± 1.3
Life span (days)	33.1 ± 1.8	30.8 ± 2.4	38.5 ± 1.6	33.8 ± 1.6
*r*_m_	0.17 ± 0.013	0.17 ± 0.013	0.15 ± 0.009	0.15 ± 0.015

## Supplementary Materials

Supplementary materials can be found at www.mdpi.com/1422-0067/18/12/2765/s1.

## Abbreviations

AOS	Allene oxide synthase
BCA	Bird cherry-oat aphid
GLVs	Green leaf volatiles
GPA	Green peach aphid
HPL	Hydroperoxide lyase
JA	Jasmonic acid
LOX	Lipoxygenase
