# Supplementary material for: Overexpression and Down-Regulation of Barley Lipoxygenase LOX2.2 Affects Jasmonate-Regulated Genes and Aphid Fecundity
Source: Int J Mol Sci. 2017 Dec 19;18(12):2765. doi: 10.3390/ijms18122765 (PMC5751364; doi:10.3390/ijms18122765)
Supplement: Supplementary file 1 [file ijms-18-02765-s001.zip › Figure S1.docx]

Figure S1:

*LOX2.2* antisense direction:

GTCGGGGCTGCAATGTGAGGGGTCGATCCAGGAGTGGCAGTGGAATAACGTGACGGCGGATGACTTTGTTGGGTCTTCACCAGCGGGGAAGATCTTGACTTCTCCTAGCAACATCTCGGTGTGGTGGTAGTTCACCAGGCGCACGGCGCCGATGGGGCCAAACGATGCCGGCACGGTAAAGATGGCTTCATATAAGTCGTAATCCTTTTCGGTTTCACGCGAGTGCTTGAGTTTGCCGGAGATTACATCATGTTCCTGCCCCGTGTTTGGATCCAACTCGGAGCTGACGAGCTCAAGGGTCAGCCATGAGCCGAGAATGAGATCGTACGCCCAATCACGGGCCCTTTCCTTCTTCTCATCCGACCACCAGAAACTCTTCATGTGCACCGACACCGTGGCCTTCATCTCGACGGTCTGCTTGAGGTCGTACTGGTGGCCCGGCTTGGCGACCGTGGTGAGGGCCGGCCCATTGACGTCGGTTAAGGTGGTGGTGGTGGTCGTGGTGGTGCTGGTGCTGCCGACCCTGGTGCGGCGGGAGTTCGTGCTCGGTTTTCGGCGAGCCTCTGCCACGAGGAACGATGCCCTCCGGCTCAACGGGACGGCACGTGCGCCTACCAAAGGCTTGGTTGCCGTCTGCAT
