# Supplementary material for: Overexpression and Down-Regulation of Barley Lipoxygenase LOX2.2 Affects Jasmonate-Regulated Genes and Aphid Fecundity
Source: Int J Mol Sci. 2017 Dec 19;18(12):2765. doi: 10.3390/ijms18122765 (PMC5751364; doi:10.3390/ijms18122765)
Supplement: Supplementary file 1 [file ijms-18-02765-s001.zip › Figure S4.docx]

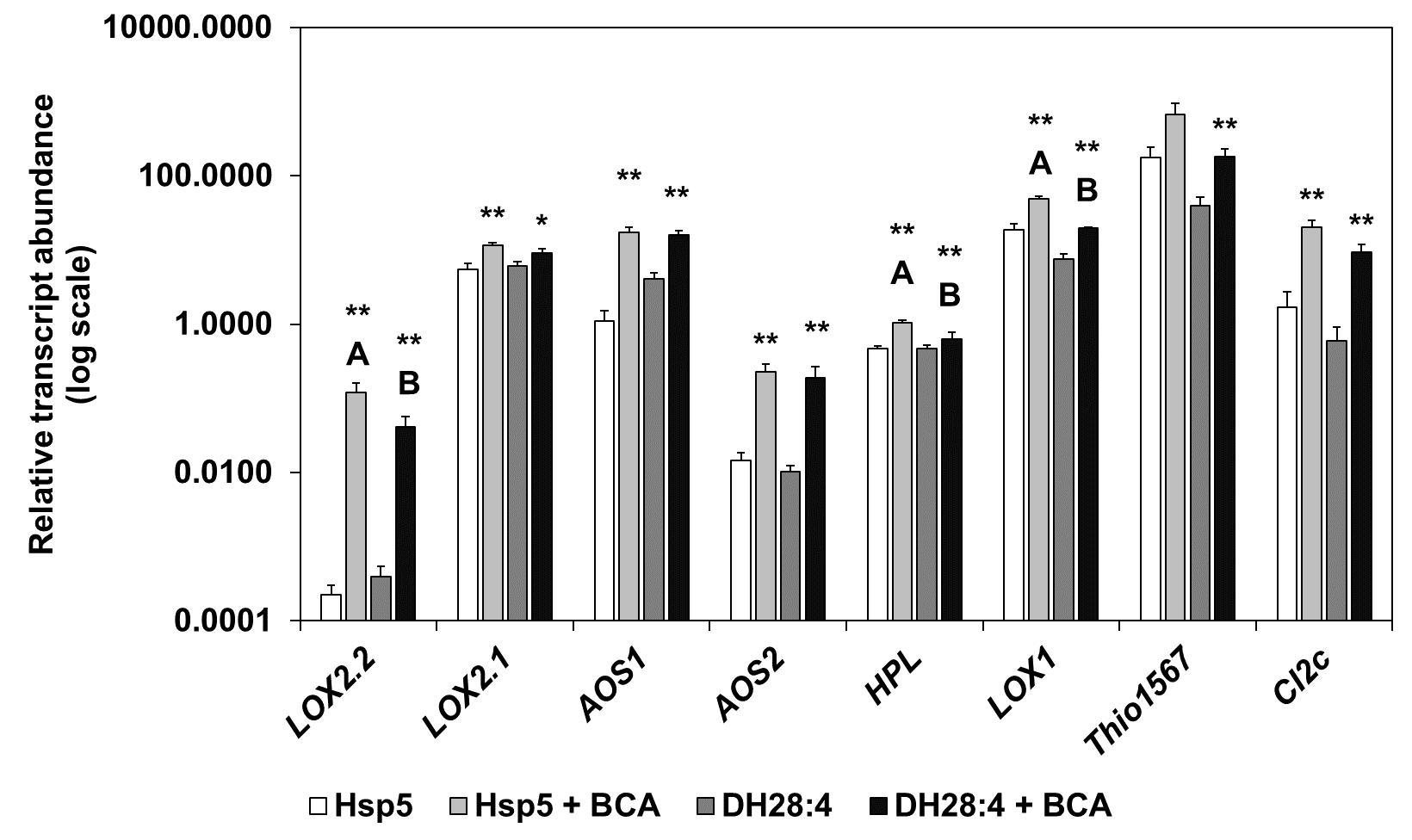


**Figure S4**. Transcript abundance of a suit of JA regulated genes in barley Hsp5 and DH28:4 with and without BCA. Primary leaves were infested with twenty adult apterous BCA during 48 h. White and light grey bars represent Hsp5, with and without aphids; dark grey and black bars represent DH28:4 with and without aphids. The transcript abundance was calculated relative to the reference genes: Hsp70 and SF427. Different letters indicate significant differences between the infested lines (Kruskal-Wallis test, p≤0.05), asterisks indicate significant difference in one genotype with or without aphids (Mann-Whitney test, *p≤0.05, **p≤0.01). Six biological replicates (with two plants each) and three technical replicates.
