# Supplementary material for: Overexpression and Down-Regulation of Barley Lipoxygenase LOX2.2 Affects Jasmonate-Regulated Genes and Aphid Fecundity
Source: Int J Mol Sci. 2017 Dec 19;18(12):2765. doi: 10.3390/ijms18122765 (PMC5751364; doi:10.3390/ijms18122765)
Supplement: Supplementary file 1 [file ijms-18-02765-s001.zip › Figure S2.docx]

**
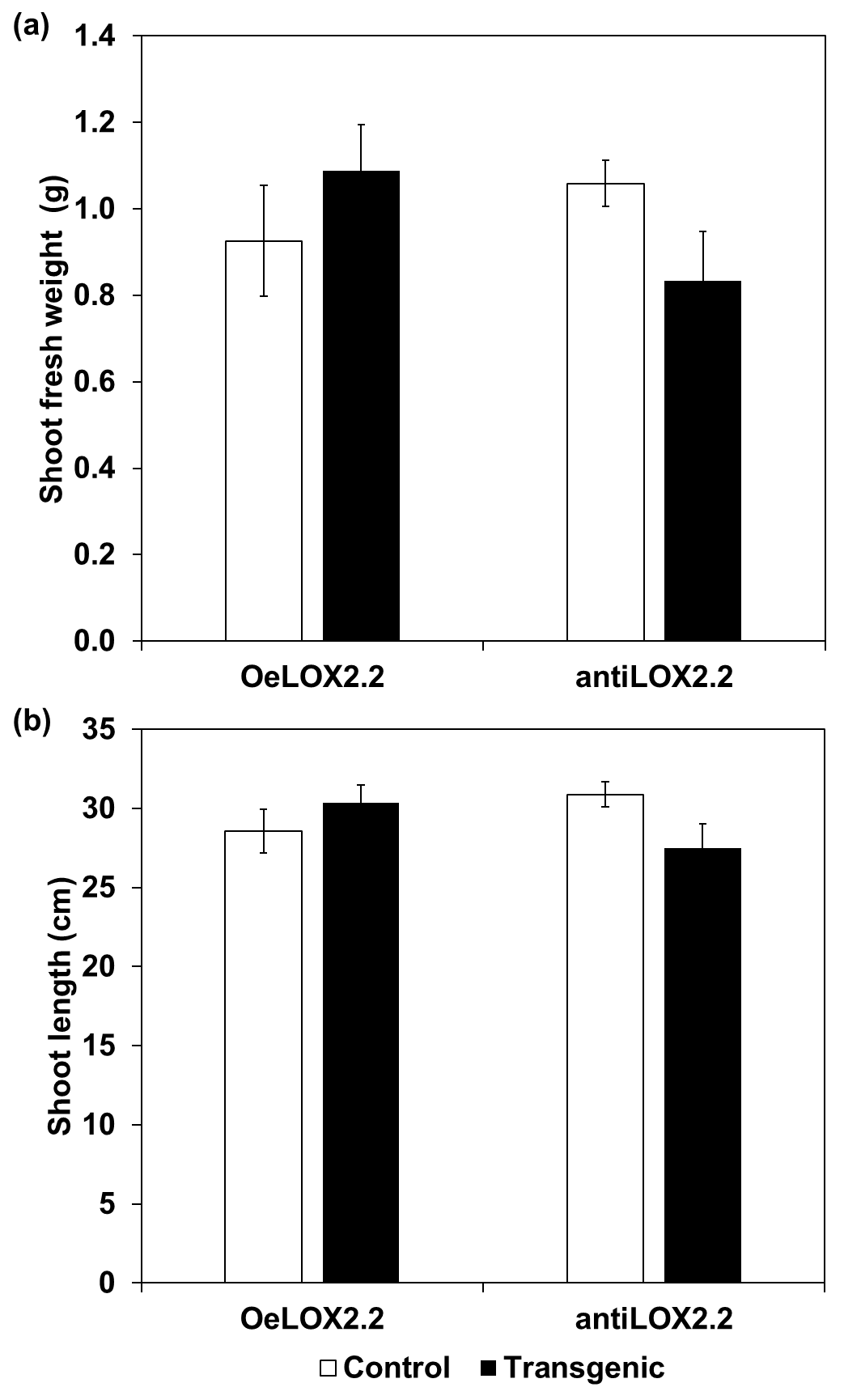
**

**Figure S2.** Fresh weight and length of control and transgenic barley shoots. (a) Shoot fresh weight. (b) Shoot length. Error bars indicate SE; n=12. Plants were 19 days old. There were no significant differences between control and transgenic lines in shoot weight (*t-*test, *p*=0.074) or length (*p*=0.056)
