# Supplementary material for: Overexpression and Down-Regulation of Barley Lipoxygenase LOX2.2 Affects Jasmonate-Regulated Genes and Aphid Fecundity
Source: Int J Mol Sci. 2017 Dec 19;18(12):2765. doi: 10.3390/ijms18122765 (PMC5751364; doi:10.3390/ijms18122765)
Supplement: Supplementary file 1 [file ijms-18-02765-s001.zip › Figure S3.docx]

**
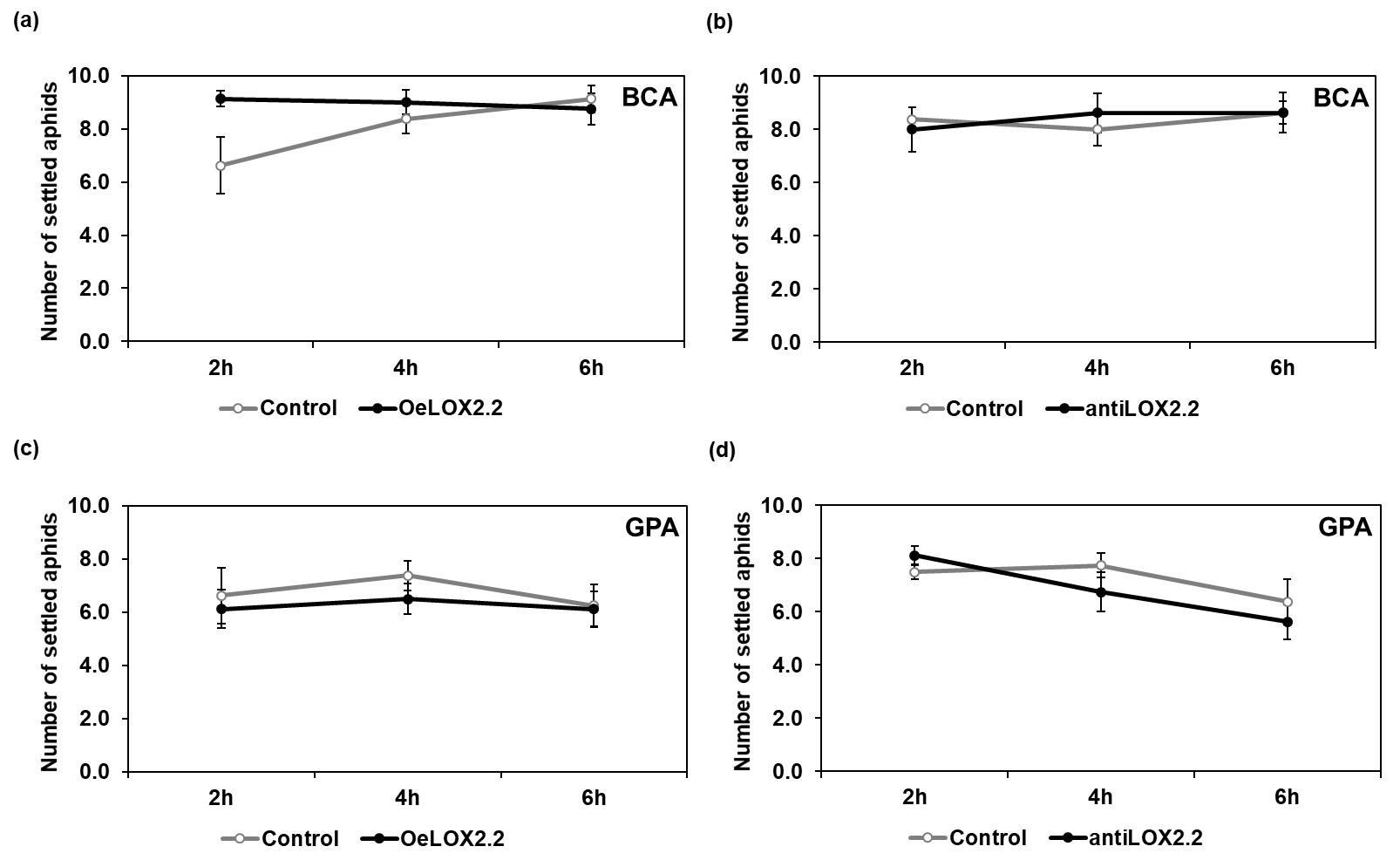
Figure S3. Number of settled BCAs and GPAs on control transgenic plants in no-choice tests**. (a, c) BCA and GPA on control and OeLOX2.2 plants. (b, d) BCA and GPA on control and antiLOX2.2 plants. Error bars indicate SE. There were no significant differences in aphid settling between control and transgenic plants at any of the time points (Mann-Whitney test, *p* > 0.05). n=8
