# Supplementary material for: Overexpression and Down-Regulation of Barley Lipoxygenase LOX2.2 Affects Jasmonate-Regulated Genes and Aphid Fecundity
Source: Int J Mol Sci. 2017 Dec 19;18(12):2765. doi: 10.3390/ijms18122765 (PMC5751364; doi:10.3390/ijms18122765)
Supplement: Supplementary file 1 [file ijms-18-02765-s001.zip › Table S1.docx]

| **Function** | **Gene abbreviation/Barley 1 contig** | **Accession number** | **Primer sequence** |
| --- | --- | --- | --- |
| Proteinase inhibitor | *CI2c/Contig50_x_at* | AJ250663.2 | F AGGTAGCGGGAAAGTCCATC  R GGTCCTGAAGTCGAGGGTCA |
| Lipoxygenase 1 | *LOX1/Contig12574_at* | AK370521 | F AAGAAGAGGAACGCCGACACCAC  R TGTCGACCGACGCACTACTCAGAC |
| Lipoxygenase 2.1 | *LOX2.1/Contig2306_s_at* | U56406.1 | F AAGATCGTGGAGCAGGTGGATGAG  R ACACACAAGGCAGCTCAAATGGAG |
| Lipoxygenase 2.2 | *LOX2.2/Contig2305_at* | AJ507212.1 | F GGTCGAAGAGTGGAACAAGGACGA  R TGCCCATCTCCATCACCGTTTTAG |
| Allene oxide synthase 1 | *AOS1/Contig3096_s_at* | AJ251304.1 | F TGTACTGGTCCAATGGGAGGGAGA  R TACGTACGTGTTGACGACCCGTTG |
| Allene oxide synthase 2 | *AOS2/Contig3097_at* | AJ250864.1 | F TACGACACCTTCACGGCCAAAGTC  R ATTTAAACAGCGTCTGCCACACCG |
| Hydroperoxide lyase | *HPL* | AJ318870.1 | F CGGTACGTGTACTGGTCCAACGG  R GGTGCACTCGAAGTCGTCGTAGC |
| Thionin | *THIO1567/Contig1567_x_at* | L36883.1 | F GCAAGAGTTGCTGCAAGAACACGA  R ATTTGGTTCACCGGATTCAGGGAG |
| Tubulin | *HvTubulin* | U40042.1 | F AGCATGAAGTGGATCCTTGG  R AGTGTCCTGTCCACCCACTC |
| Heat shock protein | *Hsp70* | (Mangelsen et al. 2010) | F CGACCAGGGCAACCGCACCAC  R ACGGTGTTGATGGGGTTCATG |
| 20S proteasome alpha subunit E | *SF427* | EY965287.1 | F ATTCCAGCCAGCGCGTAGGTAT  R CCATCACCAAGTCGCCTTTAGTAGT |

**Table S1. Primer sequences used in RT-qPCR**

Mangelsen, E. et al., 2010. Significance of light, sugar, and amino acid supply for diurnal gene regulation in developing barley caryopses. *Plant Physiology*, 153(1), pp.14–33.
